# The Influence of Body Composition Effects on Male Facial Masculinity and Attractiveness

**DOI:** 10.3389/fpsyg.2018.02658

**Published:** 2019-01-04

**Authors:** Xue Lei, Iris J. Holzleitner, David I. Perrett

**Affiliations:** ^1^School of Psychology & Neuroscience, University of St Andrews, St Andrews, United Kingdom; ^2^Institute of Neuroscience & Psychology, University of Glasgow, Glasgow, United Kingdom

**Keywords:** body composition, fat, muscle, masculinity, face preference, short-term relationship, long-term relationship, relationship context

## Abstract

Body mass index (BMI) and its facial correlates influence a range of perceptions including masculinity and attractiveness. BMI conflates body fat and muscle which are sexually dimorphic because men typically have more muscle but less fat than women. We therefore investigated the influence of facial correlates of body composition (fat mass and muscle mass) on the perception of masculinity in male faces. Women have been found to prefer more masculine looking men when considering short-term relationships compared with long-term relationships. We therefore conducted a second study of heterosexual women’s preferences for facial correlates of fat and muscle mass under long and short relationship contexts. We digitally transformed face shape simulating the effects of raised and lowered levels of body fat or muscle, controlling for each other, height and age. In Study 1, participants rated masculinity of shape-transformed male faces. The face shape correlates of muscle mass profoundly enhanced perceived masculinity but the face shape correlates of fat mass only affected the perception of masculinity in underweight to low normal weight men. In Study 2, we asked two groups of women to optimize male face images (by adjusting the shape correlates of fat and muscle) to most resemble someone they would prefer, either for a short-term sexual relationship or for a long-term relationship. The results were consistent across the two participant groups: women preferred the appearance of male faces associated with a higher muscle mass for short-term compared with long-term relationships. No difference was found in women’s preference for the face shape correlates of fat mass between the two relationship contexts. These findings suggest that the facial correlates of body fat and muscle have distinct impacts on the perception of male masculinity and on women’s preferences. The findings indicate that body composition needs to be taken into consideration in psychological studies involving body weight.

## Introduction

Research on women’s preference for male facial masculinity over the past two decades is marked by inconsistent findings. Some studies found that masculine faces were preferred by women (e.g., Rhodes et al., 2003; DeBruine et al., 2006; Feinberg et al., 2008; Little et al., 2008; Saxton et al., 2009; Jones et al., 2018), whereas other studies have reported a preference for femininity in men (e.g., Perrett et al., 1998; Penton-Voak et al., 1999; Little et al., 2002; Scott et al., 2010), and yet other studies report no overall preference for sexual dimorphism (e.g., Swaddle and Reierson, 2002; Cornwell et al., 2004).

Variability in methods has been proposed to account for the differences in results (Rhodes, 2006), yet by directly comparing commonly used methods to measure women’s preferences for male facial masculinity, DeBruine et al. (2006) found that different methods can produce similar results. Alternatively, individual differences in self-rated attractiveness, relationship status, own-health condition, exposure to violence, pathogen disgust sensitivity and resource availability might contribute to the variation in results (Holzleitner and Perrett, 2017). One factor that has been found to have a consistent effect on women’s preference for male masculinity is relationship context. Using computer graphics techniques to manipulate masculinity in male facial shape, women show a stronger preference for facial masculinity when choosing short-term partners compared to long-term partners (Little et al., 2002; Penton-Voak et al., 2003; Jones et al., 2018). In addition, this relationship context effect was more pronounced in women with partners and not found in those taking hormonal contraception pills (Little et al., 2002). This preference for masculinity in men as short-term partners has been found with a range of stimuli and modalities, including face, body, voice, and odor (Little et al., 2011a).

Sexual Strategies theory proposes that females have evolved distinct strategies to solve different problems they may encounter when pursuing a short-term or long-term relationship (Buss and Schmitt, 1993). As women’s reproductive success is restricted by the resources and protection they can obtain from men, women should prefer long-term partners who are more likely to provide paternal care, reliable resources and protection. Masculinity is perceptually associated with some negative personality traits, which might explain why women prefer less masculine men for long-term partners. Indeed, perceived facial masculinity was found to increase perceived dominance (Boothroyd et al., 2007), lower perceived paternal investment (Boothroyd et al., 2007) and decrease perceived trustworthiness (Perrett et al., 1998). Complementing these findings, several studies have found that high testosterone (an androgen contributing to male sexual dimorphism) is associated with lower likelihood of marriage, higher divorce rates and higher rates of domestic disputes (Julian and McKenry, 1989; Booth and Dabbs, 1993; Booth et al., 2000). Hence, less masculine men may be advantageous for long-term relationships.

In short-term relationships, women need not be restricted by consideration of paternal investment. Therefore, selection of partners may be guided by cues to long-term health and ‘good genes’ for immunity against currently prevalent pathogens that can be passed on to offspring (Gangestad et al., 2005). Masculinity is argued to be one cue to good genes as part of the immunocompetence handicap hypothesis (Folstad and Karter, 1992). This hypothesis states that testosterone has an immunosuppressive effect. Masculine men need a strong immune system to resist the immunosuppressive effect. Masculinity may therefore signal a strong immune system in men. Although studies examining the relationship between testosterone and immune function have produced mixed results, a recent cross-species meta-analysis revealed a medium-sized effect from experimental studies which elevate testosterone artificially and find a concomitant decline in immune function (Foo et al., 2017).

While a considerable number of studies have focused on the role of testosterone in suppressing immune function, it is relevant that testosterone has also been found to play a key role in maintaining men’s cardiovascular health. A deficiency in testosterone is associated with increased central adiposity, reduced insulin sensitivity, impaired glucose tolerance and increased cholesterol, which are all found in metabolic syndrome and type 2 diabetes and are detrimental to cardiovascular health (Kelly and Jones, 2013). Although there is debate about whether lower levels of testosterone cause cardiovascular diseases directly or whether decreased testosterone is a by-product of poor health, clinical studies have found that testosterone replacement therapy is effective in improving health in metabolic syndromes (Elagizi et al., 2018). If masculinity is heritable, masculinity may be a cue to current health and to genes for good health.

Despite the prolific research on the effect of masculine traits (e.g., faces, voices, odors) on attractiveness, few studies have explored the role that muscle plays. This is surprising considering the fact that higher muscle mass to lower fat mass is a typical masculine feature in humans (Wells, 2007) because testosterone promotes both muscle and bone growth (Mooradian et al., 1987). Thus, measures of muscle might be strong cues to masculinity. It follows that one may expect men with high muscle to be preferred by women, especially for short-term relationships, as women prefer more masculine looking men for short-term relationships. Indeed, muscular men were found to be preferred by women and have greater mating success (Frederick and Haselton, 2007).

Besides the close relationship between testosterone and muscle mass, muscularity may influence masculinity perception through its association with body size, which is also sexually dimorphic. Men on average are heavier compared to women. Indeed the faces of men with higher body mass index (BMI; weight scaled by the square of height) are perceived as more masculine than men with low BMI (Holzleitner et al., 2014). Therefore, muscular men may be perceived as masculine because they have greater weight. Since body weight is mainly composed of fat and muscle, it raises the question as to whether or not fat mass has a similar effect to muscle mass on male masculinity and attractiveness.

To our knowledge, only one study has explored the role of body composition on the perception of attractiveness in male bodies (Brierley et al., 2016). The results from this study suggest that men with levels of body fat and muscle mass in the healthy BMI range are most preferred by women. This study did not investigate the context of the attractiveness judgments. More importantly, no study has tested the effects of facial correlates of body composition (fat and muscle) on the perception of masculinity and facial attractiveness. Humans rely more heavily on facial attractiveness than physical (body) attractiveness when choosing mates (Currie and Little, 2009). In fact, when given the choice, women gave priority to men’s faces over bodies when judging dating partners for both short- and long-term relationships (Confer et al., 2010). These findings highlight the importance of investigating the effect of the facial cues to body composition on attractiveness.

In the current studies, we examine (a) the impact of facial correlates of body composition (fat and muscle) on perceived male facial masculinity, and (b) how the facial correlates of body composition influence women’s preference for male faces under short-term and long-term relationship contexts.

Considering that testosterone encourages the growth of muscle, we predict that the facial correlate of muscle mass will be positively correlated with perceived facial masculinity (Hypothesis 1). Since men are heavier than women, a heavier body no matter whether the weight is due to fat mass or muscle mass may lead to higher perceived masculinity. We thus predict the facial correlate of fat mass should also contribute positively to the perception of male facial masculinity (Hypothesis 2). Nevertheless, we expect the face shape correlate of muscle to have a larger effect on perceived facial masculinity than the face shape correlate of fat based on the stronger association between muscle and testosterone than the association between fat and testosterone (Hypothesis 3).

Regarding facial preferences, we predict that women should show a stronger preference for facial cues to increased muscle mass under a short-term relationship context compared to a long-term relationship context (Hypothesis 4). Similarly, we predict a stronger preference for facial cues to increased fat mass in short-term relationships compared to long-term relationships (Hypothesis 5). We also predict that the relationship context effect on preferences will be more apparent for the facial correlates of muscle than the facial correlates of fat (Hypothesis 6). These hypotheses about preferences follow from Hypotheses 1-3 since higher weight, particularly from muscle is expected to increase masculinity.

## Materials and Methods

### Stimuli

To examine the generalizability of findings, we included three sets of faces. One set of three-dimensional (3D) face stimuli, collected using a 3D camera and delineated with 49 landmarks using MorphAnalyser software that included scans of 50 Caucasian men (*M*_age_ ± *SD* = 21.2 ± 2.5 years, see Holzleitner and Perrett, 2016). A second set of two-dimensional (2D) images matched to the 3D scans were also available for the same 50 men (hereafter referred to as the 2D version of 3D face set). These 2D images were captured under a constant lighting condition using a Fujifilm FinePix S5Pro digital SLR camera (60 mm fixed length lens) in a booth painted with standard white paint. Facial images were captured in full color with participants’ hair pulled back. Participants, seated at a set distance from the camera and the same relative eye height to the camera, were asked to maintain a neutral expression. Faces were delineated in PsychoMorph^1^ with 189 landmarks and aligned on the left and right pupils (Tiddeman et al., 2001).

A further independent set of 2D face images was collected from 101 Caucasian male participants (*M*_age_ ± *SD* = 21.44 ± 3.33 years) who were recruited from the University of St Andrews. The participants contributing to the 3D face set and matched 2D face set did not contribute to the independent 2D face set.

### Anthropometric Measurements

Anthropometric data were acquired after removing excess clothing and footwear. Each individual’s height was measured with a tape measure (stadiometer), and body composition was measured barefoot using an electrical impedance scale (Tanita SC-330 body composition analyzer), which estimates weight, BMI, fat mass and muscle mass (lean fat-free mass). These estimations take into account information about athletic training (>10 h/week) and norms for each gender. The indicator ‘muscle mass’ refers to an estimate of the weight of fat-free mass excluding bone mass, and includes contributions from skeletal muscles, smooth muscles and cardiac muscles.

### Face Transformation

The method used to transform the face shape involves defining the difference in face shape between two groups of faces differentiated along one dimension (e.g., high/low BMI, see Holzleitner and Perrett, 2016; Batres and Perrett, 2017). The difference is then applied to individual face images.

Prototypes associated with high or low fat mass or muscle mass were first created separately for 2D and 3D faces. Prototypes were made by averaging together the nine faces for 3D face set (and matched 2D version of 3D face set) ranked the highest and lowest on the fat mass or muscle mass dimension. This allows a direct comparison between 2D and 3D faces. Since larger individuals usually have higher absolute fat mass and muscle mass than smaller individuals, fat prototypes were created with age, height and muscle mass controlled. Similarly, muscle prototypes were created with age, height and fat mass controlled. Therefore, prototypes differed only in either fat or muscle mass dimension but not in both dimensions (see Supplementary Material Table S1 for details). Similarly, we created prototypes from the 10 faces ranked highest and lowest in fat or muscle mass dimension for the independent 2D face set.

**FIGURE 1:**
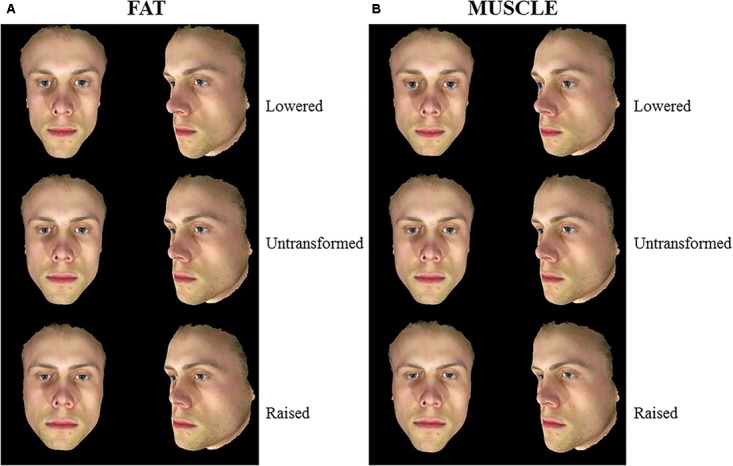
3D Male face shape associated with fat mass **(A)** and muscle mass **(B)**. Individual faces (middle) were transformed to reflect face shapes associated with less fat/muscle mass (–4 BMI units, top) or more fat/muscle mass (+4 BMI units, bottom) based on the difference in the face shape between low and high fat/muscle prototypes for the 3D face set. Front and half-profile views of the same face are displayed. The participant gave written informed consent for the publication of his image and use in the experiments.

**FIGURE 2:**
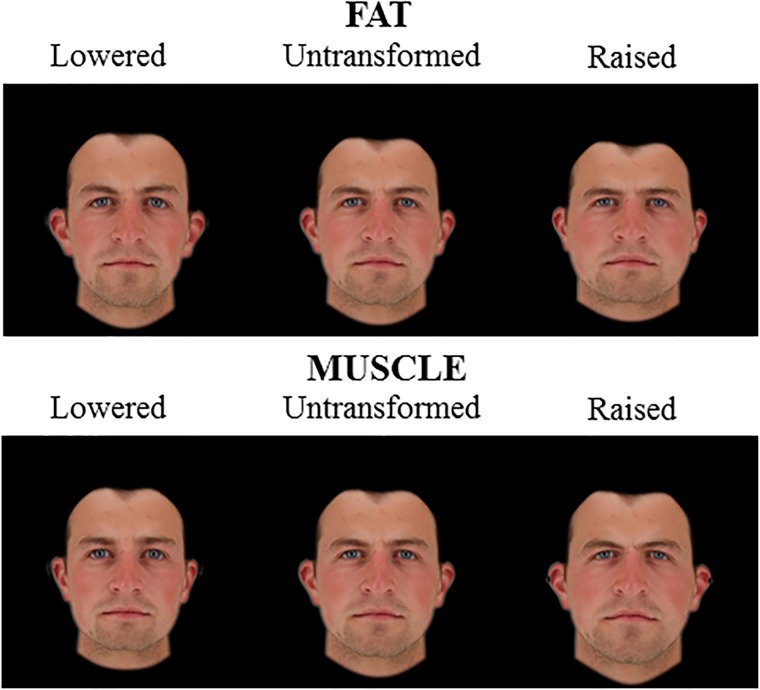
2D Male face shape associated with fat mass (top) and muscle mass (bottom). Individual faces (middle) were transformed to reflect face shapes associated with less fat/muscle mass (–4 BMI units, left) or more fat/muscle mass (+4 BMI units, right) based on the difference in the face shape between low and high fat/muscle prototypes for the 2D version of 3D face set. The participant gave written informed consent for the publication of his image and use in the experiments.

**FIGURE 3:**
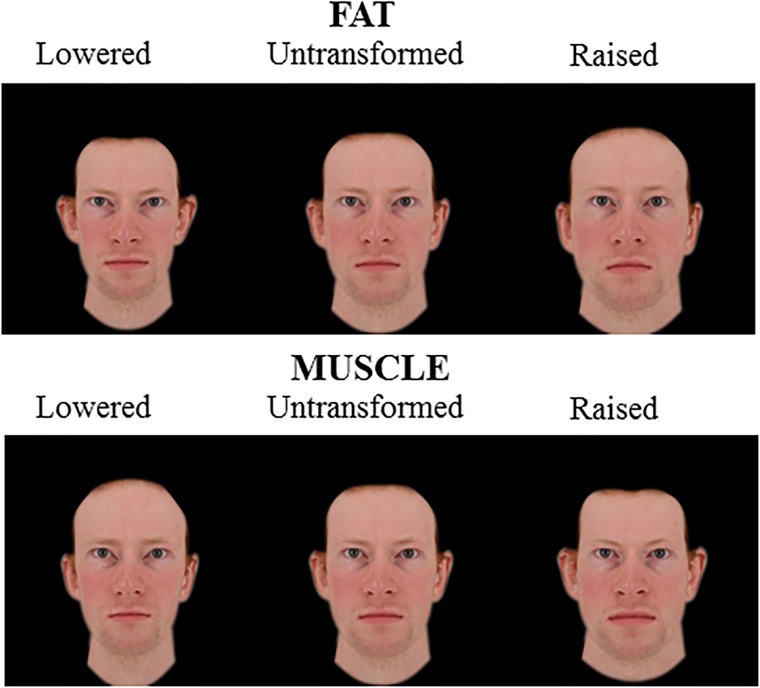
2D Male face shape associated with fat mass (top) and muscle mass (bottom). Individual faces (middle) were transformed to reflect face shapes associated with less fat/muscle mass (–4 BMI units, left) or more fat/muscle mass (+4 BMI units, right) based on the difference in the face shape between low and high fat/muscle prototypes for the independent 2D face set. The participant gave written informed consent for the publication of his image and use in the experiments.

The fat and muscle prototypes were then used to create shape transforms of five Caucasian male faces. Face shapes were transformed to visualize body composition (fat/muscle mass) differences by adding or subtracting a proportion of the facial shape differences between low and high fat/muscle prototypes. To make the fat- and muscle- transformed images comparable, facial shapes were transformed to the same magnitude in terms of BMI (±4 BMI units) in 15 steps. This process created three sets of transformed images (using 3D prototypes, 2D version of 3D prototypes and an independent set of 2D prototypes). Each set of transformed images consisted of five identities transformed to lose/gain fat/muscle mass (Figures 1–3). For 3D images, both the front view and the half-profile view were created in the transformation process. These two views were combined in one image (Figure 1).

All images were masked with the black background to display only the face and neck and to remove confounds arising from hair (DeBruine et al., 2010). 2D images were aligned to have the same pupil positions and resized to 500 × 500 pixels.

## Study 1: Facial Correlates of Body Composition and Perceived Masculinity

This study aimed at testing whether facial correlates of body composition (fat mass and muscle mass) influence perceived facial masculinity in males. We tested the following hypotheses:

(1) Faces associated with more muscle mass will be perceived as more masculine.
(2) Faces associated with more fat mass will be perceived as more masculine.
(3) The facial correlate of muscle mass has a larger impact on perceived facial masculinity than the facial correlate of fat mass.

### Methods

Ethical approval was received from University of St Andrews Ethics Committee (PS13092). Participants gave written informed consent to perform the experimental tasks.

#### Participants

Sixty-seven students from the University of St Andrews (*M*_age_ ± *SD* = 19.37 ± 3.84 years, range 18-45) including 56 females and 9 males (demographics were omitted by two participants; 51 Caucasian) completed this study.

#### Materials

Stimuli consisted of three face identities transformed to four levels (-4 BMI units, -2.3 BMI units, +2.3 BMI units, +4 BMI units) plus the untransformed image (+0 BMI units). Therefore, there was a total of 81 stimuli: 3 (face identities) × 3 (face sets: 3D face set, matched 2D version of 3D face set, independent 2D face set) × 9 [4 BMI levels × 2 dimensions (fat and muscle) + original face].

#### Procedure

Participants were asked to complete a demographic questionnaire (age, sex, ethnicity, and sexual orientation). Then faces were presented one at a time in three blocks (each block consisted of a set of faces with muscle and fat transform). Both the order of the trials within blocks and the three blocks were completely randomized. Participants were asked to rate the masculinity (“Please indicate how masculine you perceive this man to be”) of each stimulus face by dragging the cursor on a sliding bar with anchors (1 = least masculine and 7 = most masculine). The starting point of the cursor along the bar was randomized. There was no time limit to make judgments. The next face was shown only after the participant had adjusted the slider and clicked for the next trial.

#### Statistical Analysis

For each stimulus type, the mean ratings were calculated across face identities for each participant. The consolidated data were further analyzed in SPSS 24.0 three-way analysis of variance (ANOVA) was run, with the transform dimension (fat/muscle) and the transform level (five levels: -4 BMI units, -2.3 BMI units, no change, +2.3 BMI units, +4 BMI units) included as the independent variables. Face set (three sets) was included as an additional independent variable to determine if results were consistent across the different samples of faces.

### Results

A three-way ANOVA was run to test the transformation attributions made to fat and muscle mass across the three face sets. The results showed non-significant main effects of the transform dimension [*F*(1,66) = 0.44, *p* = 0.507, η^2^ = 0.007] and face sets [*F*(2,132) = 0.94, *p* = 0.392, η^2^ = 0.014] on masculinity rating, but a significant main effect of transform level [*F*(4,264) = 74.80, *p* < 0.001, η^2^= 0.531] (see Table 1). As face shape simulated heavier individuals (higher BMI), the masculinity ratings increased. The interaction between transform dimension and face set was not significant [*F*(2,132) = 0.41, *p* = 0.665, η^2^ = 0.006] but a significant interaction was found between transform dimension and transform level [*F*(4,264) = 24.75, *p* < 0.001, η^2^= 0.273], reflecting a greater impact of muscle transform compared with fat transform on masculinity.

**Table 1:** Descriptive statistics of mean masculinity ratings (1-7) (SD) for three sets of faces transformed in fat mass and muscle mass dimensions at five BMI levels.

	-4 BMI	-2.3 BMI	0	+2.3 BMI	+4 BMI
	
	M (SD)	M (SD)	M (SD)	M (SD)	M (SD)
	**Fat**				
3D face set	3.62 (0.92)	3.93 (0.70)	4.17 (0.70)	4.21 (0.80)	4.26 (0.84)
2D version of 3D face set	3.49 (0.92)	3.84 (0.83)	4.08 (0.76)	4.23 (0.86)	4.42 (1.01)
Independent 2D face set	3.75 (0.99)	4.00 (0.83)	4.27 (0.82)	4.22 (0.96)	4.32 (1.08)
	**Muscle**				
3D face set	3.47 (0.83)	3.91 (0.63)	4.17 (0.70)	4.27 (0.79)	4.54 (0.88)
2D version of 3D face set	3.10 (0.91)	3.74 (0.80)	4.08 (0.76)	4.42 (0.89)	4.68 (1.06)
Independent 2D face set	3.39 (0.99)	3.66 (0.85)	4.27 (0.82)	4.53(0.92)	4.73(1.12)


There was a significant interaction between face set and transformed level [*F*(8,528) = 2.61, *p* = 0.008, η^2^= 0.038]. Further, the three-way interaction among transform dimension, transform level, and face set was significant [*F*(8,528) = 2.17, *p* = 0.028, η^2^= 0.032]. To understand the three-way interaction, we conducted two-way ANOVA separately for each face set.

#### 3D Face Set

**FIGURE 4:**
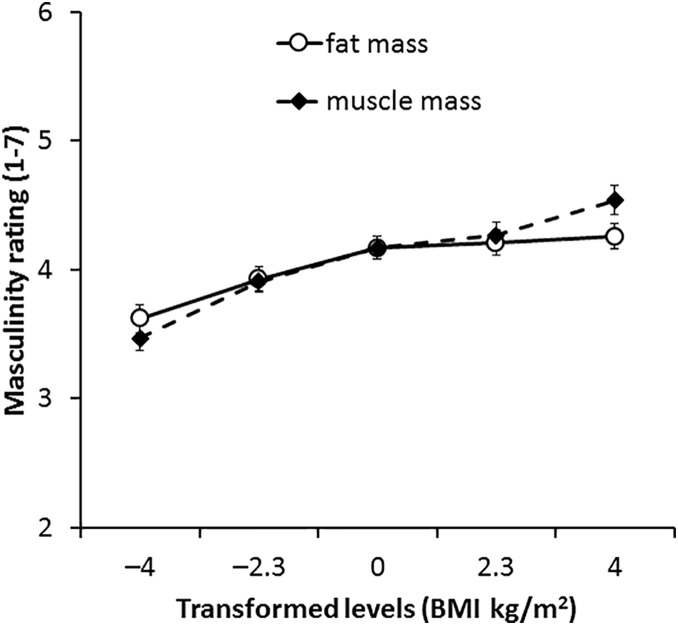
Average masculinity ratings for faces transformed with the face shape correlates of fat and muscle mass for the 3D face set. Error bars represent the standard errors.

For 3D faces, the main effect of the transform dimension was non-significant [*F*(1,66) = 1.36, *p* = 0.252, η^2^= 0.020]. There was a significant main effect of transform level [*F*(4,264) = 31.17, *p* < 0.001, η^2^= 0.321], which was qualified with an interaction between transform dimension and transform level [*F*(4,264) = 4.40, *p* = 0.002, η^2^= 0.062, see Figure 4]. Paired-samples *t*-tests showed that significant increases in masculinity ratings occurred between all levels of muscle transform (*p* ≤ 0.004 each comparison) except between 0 and +2 BMI units (*p* = 0.186). By contrast, there were no significant increases in masculinity ratings for fat transform above normal weight (0, +2.3, and +4 BMI units, *p* ≥ 0.337 each comparison). There were significant decreases in masculinity ratings between faces associated with decreased fat mass compared to increased fat mass (*p* ≤ 0.005 each comparison). These findings provide further support for our Hypothesis 3 that the facial correlate of muscle mass increases perceived facial masculinity more than the facial correlate of fat mass.

#### 2D Version of 3D Face Set

**FIGURE 5:**
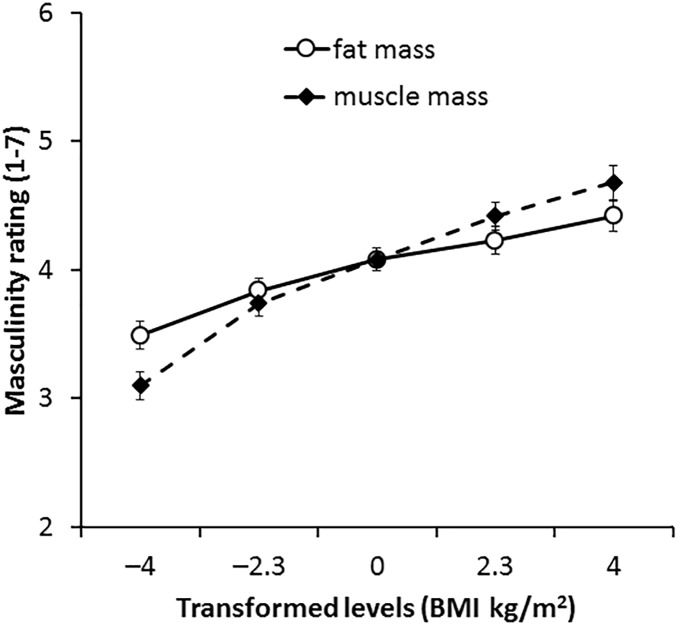
Average masculinity ratings for faces transformed with the face shape correlates of fat and muscle mass for the 2D version of 3D face set. Error bars represent the standard errors.

For the 2D version of the 3D face set, there was no main effect of transform dimension [*F*(1,66) = 0.05, *p* = 0.833, η^2^= 0.001]. The main effect of transform level was significant [*F*(4,264) = 50.85, *p* < 0.001, η^2^= 0.435] but was qualified by a significant interaction between transform dimension and transform level [*F*(4,264) = 8.63, *p* < 0.001, η^2^= 0.116, see Figure 5]. Paired-samples *t*-tests showed an increase in muscle mass by ∼2 BMI units significantly increased masculinity ratings throughout the range (-4 to +4 BMI units, *p* ≤ 0.002 each comparison). Significant increases in masculinity ratings with fat mass transform were seen in most comparisons (*p* ≤ 0.014 each comparison) but no significant increases were seen in comparisons between faces associated with increased fat mass [0 vs. +2.3 BMI units (*p* = 0.170) and +2.3 vs. +4 BMI units (*p* = 0.070)]. These findings are again in line with our prediction that facial correlates of both fat mass and muscle mass positively influence perceived facial masculinity but that also the facial correlate of muscle mass has a larger impact on masculinity.

#### Independent 2D Face Set

For face transforms based on the independent 2D face set, the main effect of the transform dimension was non-significant [*F*(1,66) = 0.02, *p* = 0.888, η^2^ = 0.000]. A significant main effect of transform level [*F*(4,264) = 34.89, *p* < 0.001, η^2^= 0.346] reflected faces associated with increased mass (fat or muscle) being considered more masculine.

**FIGURE 6:**
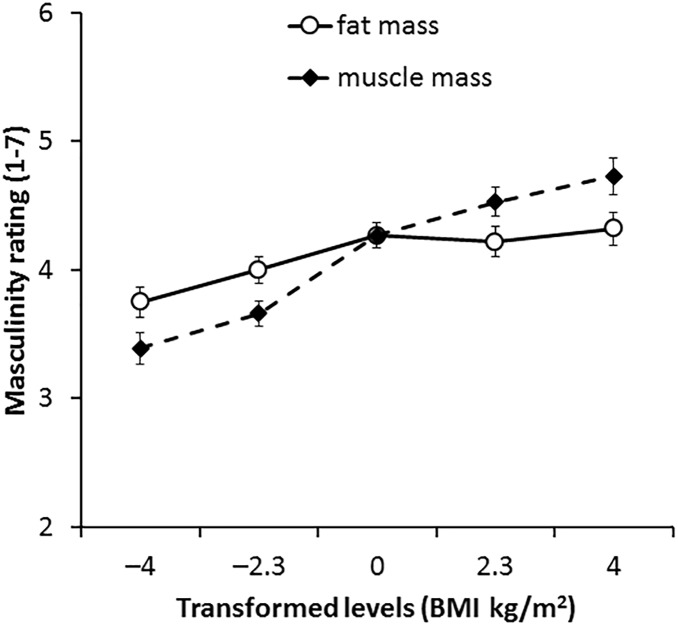
Average masculinity ratings for faces transformed with the face shape correlates of fat and muscle mass for the independent 2D face set. Error bars represent the standard errors.

The interaction between transform dimension and transform level was significant [*F*(4,264) = 15.82, *p* < 0.001, η^2^= 0.193, see Figure 6]. This interaction reflects a greater impact of muscle compared with fat on masculinity ratings. Paired-samples *t*-tests showed that participants rated faces with higher muscle mass significantly more masculine for comparisons between all five levels (*p* ≤ 0.017 each comparison). In contrast, a significant increase in masculinity ratings for faces associated with higher fat mass was evident only for comparisons between faces with decreased fat mass (-4 BMI units, -2.3 BMI units) and the other levels (*p* ≤ 0.046 each comparison). There were no significant differences in masculinity ratings for fat transforms 0, +2.3, or + 4.3 BMI units (*p* ≥ 0.270 each comparison). As fat mass increased from low to normal weight, masculinity increased, but for gain in the fat level above normal weight, there was no significant change in masculinity ratings. These findings support our hypothesis that the facial correlate of muscle mass enhances perceived facial masculinity more than the facial correlate of fat mass.

The interaction between face set, transform dimension and transform level arises from the relative size of the muscle and fat transforms across the three face sets, with the fat and muscle differences being most subtle in the 3D face set though the pattern is similar for each face set.

### Discussion

As expected, facial correlates of fat mass and muscle mass both positively affected perceived facial masculinity in men. The results are consistent with Holzleitner et al. (2014) findings of heavier men being perceived as more masculine. As we hypothesized, muscle mass enhances the perception of masculinity more than fat mass. Specifically, increasing the face shape correlate of muscle mass resulted in higher ratings of facial masculinity across the full weight range (BMI range 18-26). By contrast, increasing the face shape correlate of fat mass only raised masculinity rating from low to normal weight (BMI = 18-22). Further increases in fat mass above normal weight (BMI = 22) had little or no impact on the perception of masculinity. These results imply that the effect of fat on masculinity is more prevalent in men with underweight to normal weight bodies.

## Study 2: Attraction to the Facial Correlates of Body Composition

Study 1 found that facial correlates of both fat mass and muscle mass contribute to perceived facial masculinity, which has been found to affect the perception of attractiveness. In this part of the study, we tested the relationship between facial correlates of body composition and facial attractiveness.

As discussed before, higher levels of masculinity are preferred by women more for short-term relationships than for long-term relationships. Hence, we measured heterosexual women’s preferences for facial correlates of body composition in male faces under short-term and long-term relationship contexts. Given the findings above that the facial correlate of muscle mass increases perceived facial masculinity, we predicted that *women would show a stronger preference for the facial correlate of muscle mass in a short-term rather than a long-term relationship context* (Hypothesis 4). Regarding fat mass, in the introduction we hypothesized that women would show a stronger preference for higher fat mass in short-term relationships than in long-term relationships. In the light of the masculinity ratings we found in Study 1, this hypothesis should be modified. We can now hypothesize that *if women show an overall preference for men with a BMI < 22, we predict women will prefer a face shape associated with more fat mass for a short-term relationship in comparison to a long-term relationship* (Hypothesis 5a). Conversely, we predict that *women will not shift their preference for the facial correlate of fat mass between short-term and long-term relationships if they prefer men with a BMI > 22* (Hypothesis 5b). Nevertheless, we predict the preference shift between short-term and long-term contexts will be more apparent for the facial correlate of muscle mass than the facial correlate of fat mass (Hypothesis 6).

This study was initially administered with Study 1 as a single experiment consisting of two tasks (masculinity rating and preference) for University students, with the preference task executed before the masculinity task. Considering the students are highly homogeneous groups due to their age and educational background, the study was repeated in a more heterogeneous group to test the generalizability of findings. Hence, we recruited another group of participants through the online recruitment platform, Amazon MTurk.

### Methods

Ethical approval was received from University of St Andrews Ethics Committee (PS13176 and PS13092). Participants gave written informed consent to perform the experimental tasks.

#### Participants

For the student group, 63 heterosexual female participants (*M*_age_ ± *SD* = 18.94 ± 2.17, range 18–35 years; 48 Caucasian) completed this study after exclusion of those without demographic information (age, sex, ethnicity, and sexual orientation) or who reported to be homosexual or males. For the MTurk workers group, 58 heterosexual women (*M*_age_ ± *SD* = 32.09 ± 6.68, range 22–45 years; 43 Caucasian) completed this study after exclusion using the same criteria as the students’ group and an additional exclusion age criterion. Ten women over age 45 years were additionally excluded as our prediction was based on the assumption that the key benefit women gain from short-term relationships concerns potential reproductive success. MTurk participants were paid $3 for their time.

#### Materials

The stimuli consisted of face images transformed as described above. For each face identity, 15 images were produced spanning the transformation ±4 BMI units on fat mass and muscle mass dimensions. The 15 images were presented as an interactive continuum. For MTurk workers, a total of 30 face continua: 5 face identities × 2 dimensions (fat/muscle) × 3 face sets (3D face set, 2D version of 3D face set, independent 2D face set) were presented twice in separate trial blocks asking about preferences for a short-term sexual relationship and long-term relationship. For the student group, the three face identities were used. Thus, 18 face continua: 3 identities × 2 dimensions (fat/muscle) × 3 face sets (3D face set, 2D version of 3D face set, independent 2D face set) were presented in each of two trial blocks.

#### Procedure

At the beginning of this study, participants were asked, “ Please indicate the sex of face that you would like to see (as a sexual partner)” (Note: female faces were also given as an option for heterosexual males, homosexual and bisexual female participants to view, but data from these faces are not analyzed here). The participants’ demographic information (age, sex, ethnicity, and sexual orientation) was collected in an initial questionnaire. Then participants were presented with the stimuli twice in two blocks. They were asked to adjust the slider underneath each stimulus to make the face most resemble someone they would find attractive as a short-term (sexual) partner and as a long-term partner in two separate blocks. The order of the tasks was counterbalanced. Trials with 2D and 3D face stimuli were also grouped in two separate sub-blocks. The order of sub-blocks and the presentation order within each sub-block was randomized. The scroll direction to change the face shape was randomized across trials. The next image would only be shown when participants adjusted the slider and clicked the submit button. For each trial, the BMI level chosen by each participant was saved.

Instructions were given prior to tasks as follows (a) Short-term (sexual) relationship: “Please change the face to most resemble someone you would find attractive for a SHORT-TERM (sexual) relationship.” (b) Long-term relationship: “Please change the face to most resemble someone you would find attractive for a LONG-TERM relationship.”

#### Statistical Analysis

The dependent variable was the transform level that was most preferred (expressed as a BMI equivalent). The data for the students group and MTurk group were analyzed separately in SPSS 24.0.

### Results

#### Student Group

**FIGURE 7:**
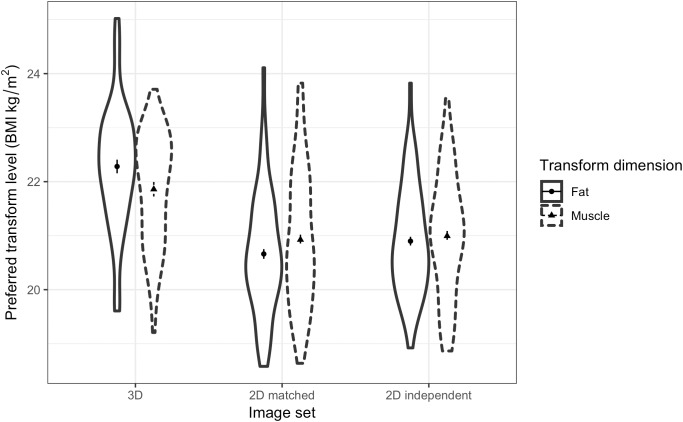
Violin plots showing the distribution of female students’ preferences for the facial correlates of fat mass and muscle mass in men. The vertical axis represents the associated BMI of the most preferred faces chosen by the students in short-term and long-term relationship contexts. The error bars represent the standard errors and the symbols indicate means.

A three-way ANOVA was run to test women’s preference for facial correlates of fat mass and muscle mass in different relationship contexts and across the three face sets. The results showed a non-significant main effect of fat/muscle transform dimension [*F*(1,62) = 3.18, *p* = 0.079, η^2^= 0.049]. As expected, a significant main effect of context [*F*(1,62) = 9.26, *p* = 0.003, η^2^= 0.130] was found, with participants preferring faces of heavier men (with fat mass or muscle mass) for a short-term relationship (*M* = 21.42, *SD* = 1.15) rather than a long-term relationship (*M* = 20.98, *SD* = 0.90). In addition, there was a significant main effect of face set [*F*(2,124) = 107.37, *p* < 0.001, η^2^= 0.634, see Figure 7]. Although we did not expect to find a main effect of the face set, the paired-samples *t*-tests suggest that the effect might simply be due to participants choosing heavier faces in the 3D face set compared with the other two 2D face sets. Paired-samples *t*-tests showed that participants choose heavier faces for the 3D face set (*M* = 22.14, *SD* = 1.07) compared with the 2D version of 3D face set (*M* = 20.67, *SD* = 0.99) [*t*(62) = 12.02, *p* < 0.001] and the independent 2D face set (*M* = 20.80, *SD* = 0.93) [*t*(62) = 10.88, *p* < 0.001].

**FIGURE 8:**
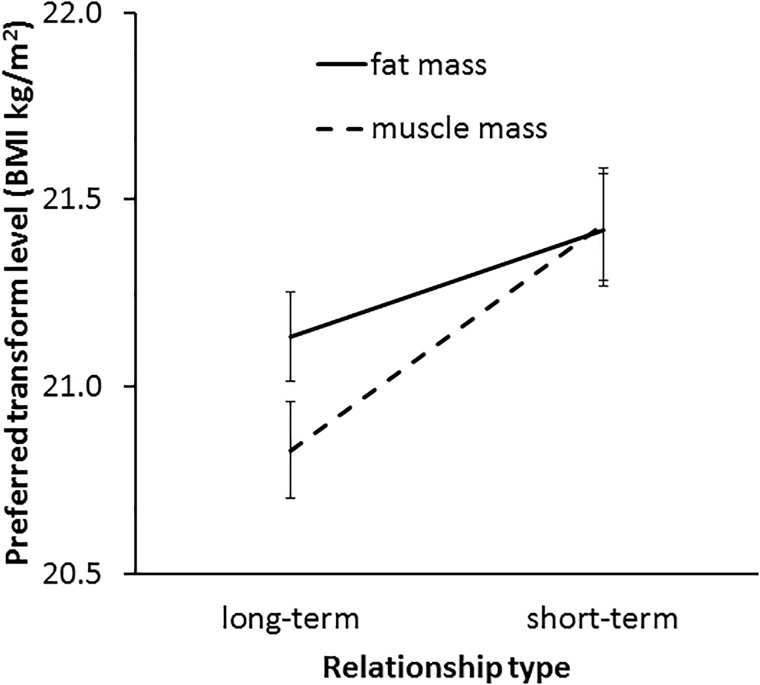
The interaction between relationship type (short-term and long-term) and preferred facial correlates of body composition (fat mass and muscle mass) in student participants. The vertical axis represents the associated BMI of the most preferred faces. Error bars represent standard errors.

In line with our Hypothesis 6, a significant interaction was found between transform dimension and context [*F*(1,62) = 4.73, *p* = 0.034, η^2^= 0.071, see Figure 8]. This result indicates a greater effect of muscle than fat on preference in the two contexts. As expected, paired-samples *t*-tests showed that a higher level of facial correlate of muscle mass was preferred in a short-term (*M* = 21.43, *SD* = 1.22) rather than a long-term (*M* = 20.83, *SD* = 1.07) relationship [*t*(62) = 3.49, *p* = 0.001]. By contrast, there was a non-significant trend for a difference between preference for the facial correlate of fat mass in short-term (*M* = 21.42, *SD* = 1.23) and long-term (*M* = 21.13, *SD* = 0.96) [*t*(62) = 1.86, *p* = 0.068] relationships, which provides limited support for Hypothesis 5a.

The three-way interaction (transform dimension × relationship context × face set) was non-significant [*F*(2,124) = 0.33, *p* = 0.719, η^2^= 0.005]. Since the interaction between fat and muscle transform and relationship context was found to be significant and independent of the face set, it was not necessary to analyze the data further for each face set separately. Thus, our main prediction was borne out across the three face sets.

Finally, one-sample *t*-tests compared the preferred BMI (average across the three face sets) with a BMI of 22 (the average of the original starting BMI of the face stimuli) to test whether women show a general preference toward a lower or higher than normal weight. Significant decreases in preferred BMI below 22.0 were found, reflecting a reduction of fat mass and muscle mass for both short-term [fat mass: *M* = 21.42, *t*(62) = -3.78, *p* < 0.001; muscle mass: *M* = 21.43, *t*(62) = -3.70, *p* < 0.001] and long-term [fat mass: *M* = 21.13, *t*(62) = -7.18, *p* < 0.001; muscle mass: *M* = 20.83, *t*(62) = -8.72, *p* < 0.001] relationships.

#### MTurk Workers

**FIGURE 9:**
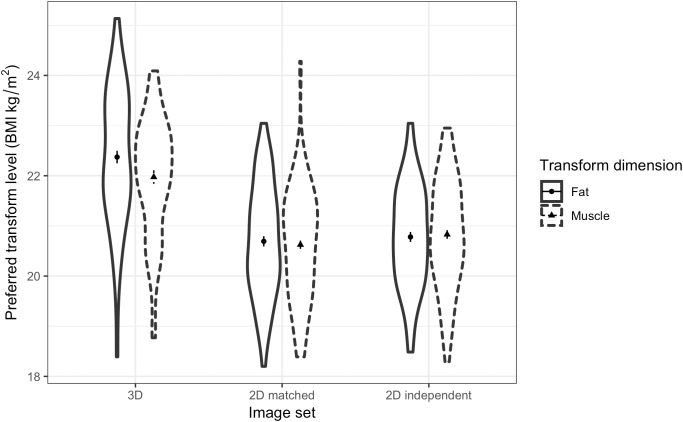
Violin plots showing the distribution of MTurk women’s preferences for the facial correlates of fat mass and muscle mass in men. The vertical axis represents the associated BMI of the most preferred faces chosen by the women in short-term and long-term contexts. The error bars represent the standard errors and the symbols indicate means.

Similarly, a three-way ANOVA was run to test MTurk women’s preference for men’s facial correlates of fat and muscle mass across relationship contexts. The results showed non-significant main effects of transform dimension [*F*(1,57) = 0.06, *p* = 0.808, η^2^= 0.001] and context [*F*(1,57) = 1.31, *p* = 0.258, η^2^= 0.022]. A significant main effect of face set was found [*F*(2,114) = 71.58, *p* < 0.001, η^2^= 0.557, see Figure 9]. Similar to the student group, paired-samples *t*-tests showed that participants chose heavier faces (with higher fat mass or muscle mass) with the 3D face set (*M* = 22.07, *SD* = 0.89) compared with the 2D version of 3D face set (*M* = 20.79, *SD* = 1.10) [*t*(57) = 8.89, *p* < 0.001] and the independent 2D face set (*M* = 20.95, *SD* = 0.93) [*t*(57) = 8.68, *p* < 0.001]. Unlike the results from the student group, MTurk participants preferred slightly heavier faces for the independent 2D face set compared to the 2D version of 3D face set [*t*(57) = -2.65, *p* = 0.010].

**FIGURE 10:**
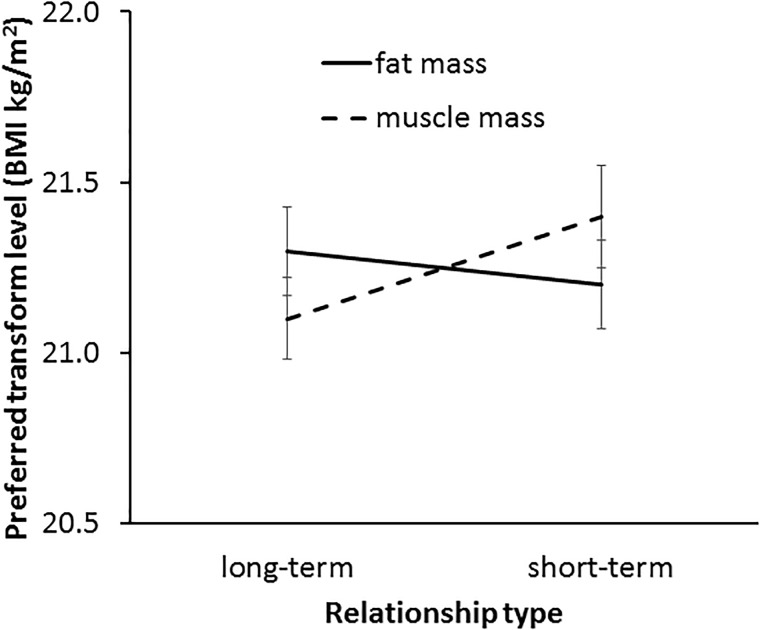
The interaction between relationship context (short-term vs. long-term relationship) and preferred facial correlates of body composition (fat mass and muscle mass) in MTurk participants. The vertical axis represents the associated BMI of the most preferred faces. Error bars represent standard errors.

In line with our Hypothesis 6, a significant interaction was found between fat and muscle transform dimension and relationship context [*F*(1,57) = 7.36, *p* = 0.009, η^2^= 0.114, see Figure 10]. Paired-samples *t*-tests results suggest that MTurk women showed a stronger preference for the facial correlate of muscle mass in short-term relationships (*M* = 21.42, *SD* = 1.12) compared with long-term relationships (*M* = 21.10, *SD* = 0.95) [*t*(57) = 2.33, *p* = 0.024] but those women did not differ in their preference for the facial correlate of fat mass between short-term (*M* = 21.24, *SD* = 0.99) and long-term relationships (*M* = 21.32, *SD* = 0.96) [*t*(57) = 0.70, *p* = 0.488]. Further, the three-way interaction (transform dimension × relationship context × face set) was non-significant [*F*(2,114) = 1.52, *p* = 0.224, η^2^= 0.026], indicating that the interaction between fat/muscle transform and relationship context was consistent across the three face sets.

One-sample *t*-tests compared the preferred BMI transform level (average across the three face sets) to a BMI of 22 (the average of the original starting BMI of the face stimuli). MTurk participants preferred a BMI significantly reduced from a BMI of 22 for both fat mass and muscle mass in short-term [fat mass: *M* = 21.24, *t*(57) = -5.82, *p* < 0.001; muscle mass: *M* = 21.42, *t*(57) = -3.96, *p* < 0.001] and long-term [fat mass: *M* = 21.32, *t*(57) = -5.37, *p* < 0.001; muscle mass: *M* = 21.10, *t*(57) = -7.17, *p* < 0.001] relationships.

### Discussion

This study investigated heterosexual women’s preferences for men’s facial correlates of body composition under different relationship contexts. In line with our Hypothesis 4, women showed a stronger preference for faces associated with higher muscle mass in a short-term relationship compared with a long-term relationship. In contrast, women did not shift their preference for the facial correlate of fat mass between short-term and long-term relationships even though their overall preference lay in the low end of normal weight (BMI∼21 kg/m^2^).

## General Discussion

The present study had two aims: first, to investigate the effect of facial correlates of body composition (fat mass and muscle mass) on the perceived facial masculinity of men and second to investigate the effect of facial correlates of body composition on women’s preferences in different relationship contexts. Ratings of masculinity supported our hypotheses that both facial correlates of fat mass and muscle mass positively affect perceived facial masculinity. While the facial correlate of muscle mass had a pronounced effect on perceived masculinity, the effect of the facial correlate of fat mass increased masculinity only in underweight to lower normal weight men. In interactive preferences tests where women optimized the shape of a male face, we found that there is a context shift in preferences with women preferring facial correlates of higher muscle mass for a short-term relationship compared to a long-term relationship. By contrast, we found that women do not shift their preference for the facial correlate of fat mass between short-term and long-term relationships.

### Attribution to Perceived Facial Masculinity

The results from Study 1 supported our predictions that facial correlates of body composition influence perceived facial masculinity. In line with Holzleitner et al. (2014) findings, the facial cues to higher body weight (BMI) increase perceived facial masculinity of male faces. The results extend previous findings that ‘facial adiposity’ (weight perceived from the facial appearance) is positively associated with perceived masculinity in under to normal weight men but not in overweight or obese men (Phalane et al., 2017). It should be noted that the definition of facial adiposity in Phalane et al.’s (2017) study was a measure of the weight perceived from the face. Hence, their perceived adiposity measure will include two components, namely weight from fat and weight from muscle. Phalane et al.’s (2017) results indicate a quadratic relationship between perceived facial adiposity and masculinity. By distinguishing the facial correlates of fat and muscle, we find a quadratic relationship between fat and masculinity, but a linear relationship between muscle and masculinity. Hence our study shows that the findings of Phalane et al. (2017) are likely to reflect the facial correlate of fat. Our findings indicate that the muscle and fat components should be treated separately in future work on facial perception.

Importantly, our results were consistent across the three face sets employed. Although the relationship between the facial correlate of fat mass and masculinity was slightly different between the 2D version of 3D face set and the other two face sets, the facial correlate of muscle mass was found to have a larger impact on perceived masculinity across all three sets of faces.

The distinct effects of fat mass and muscle mass on perceived facial masculinity might reflect the sex differences in body physique because men are generally heavier in body weight and have more muscle mass than women (Wells, 2007). Indeed, fat-free muscle mass are even more sexually dimorphic than differences in body weight (Lassek and Gaulin, 2009). Hence, heavier men with higher muscle mass have attributes associated with higher sexual dimorphism and should be seen as more masculine. Indeed, this is what we found in the first part of our study. Although men on average have greater weight compared to women, the weight difference is mainly due to the higher muscle mass that men possess. Hence, the excess fat mass does not make male faces more masculine but decreased weight, whether due to loss of fat mass or loss of muscle mass, decreases men’s perceived masculinity.

It is also possible that the facial correlates of muscle serve as a cue to testosterone levels and thus enhance masculinity perception more than the facial correlate of fat mass. In fact, increased testosterone levels during puberty cause growth of jaw, brow, chin and nose (Marečková et al., 2011). As a result, adult male faces have a relatively longer and broader lower jaw, higher brow ridges, thinner cheeks and more prominent cheekbones compared to adult women (Little et al., 2011b). The perceptual studies here provide further evidence that the face shape correlates of fat mass and muscle mass are distinct in men. Holzleitner and Perrett (2016) found that observers were able to distinguish the face shape correlates of fat mass and muscle mass using 3D facial stimuli. Here, we find further distinctions for the fat and muscle aspects of body composition for both 2D and 3D facial stimuli. A visual adaptation study also suggested that body fat and muscle are processed independently in the brain (Sturman et al., 2017). The face shape correlates of muscle may not only provide cues to body composition and physique but also may provide a cue to testosterone levels, and hence influence masculinity perception.

Taken together, we have shown that the perception of male facial masculinity is not only based on the cues to body weight. More importantly, muscularity is the aspect of the body composition that has greatest influence on facial masculinity perception.

### Context Shifts in Preferences for Facial Masculinity

Study 2 indicates that women’s preference for male face shape is dependent on context: we found that women preferred faces associated with a higher muscle mass for short-term relationships rather than long-term relationships but that women do not show different preferences for facial cues to fat mass between short- and long-term relationships.

Our findings appear to be in line with the good genes hypothesis, which argues that women are attracted to indicators signaling heritable aspects of immunity and health when seeking short-term partners (Gangestad and Simpson, 2000; Gangestad et al., 2005). We note that the contextual differences in preferences are also consistent with an alternative interpretation that the preference difference might reflect avoidance of negative characteristics associated with higher muscularity in long-term relationships. Previous studies have revealed that men with high testosterone levels and more fat-free mass (greater muscle mass) report having a larger number of sex partners, indicating that these men might devote more effort in mating relative to parenting (Peters et al., 2008; Lassek and Gaulin, 2009). Further, other studies show that men with high testosterone levels are less likely to get married and more likely to get divorced (Julian and McKenry, 1989; Booth and Dabbs, 1993; Booth et al., 2000). Hence, male faces that reflect high levels of androgen-mediated traits may be less preferred by women in a long-term relationship because of the associated behavioral traits that are inconsistent with paternal investment.

This interpretation may also account for why women do not show different preferences for the facial correlate of fat mass between the two relationship contexts. Although we predicted facial cues to higher fat mass would be preferred for short-term relationships because higher fat mass contributes to facial masculinity (at least in low weight men), the masculinity perception contributed by the facial correlate of fat mass, however, is not testosterone dependent. Therefore, despite the fact that faces associated with higher fat mass are perceived to be more masculine, the same facial cues to fat mass are not necessarily associated with the undesirable testosterone-mediated traits. Consequently, women do not need to shift their preference between short- and long-term relationships since there are no (or fewer) associated costs with preferring masculinity that derives from slightly higher fat mass. Therefore, the relationship context preference differences that we find may reflect women’s reluctance to choose very muscular men who appear unsuitable as long-term partners. Future studies investigating the perception of personality traits from facial cues to fat mass and muscle mass may provide better understandings for the context shifts.

It worth mentioning that women generally prefer faces reflecting low fat mass and muscle mass under both contexts. The associated BMI of the most preferred face was significantly reduced compared with the original starting BMI of the facial stimuli (namely BMI of 22.0 kg/m^2^). This suggests that men with low-normal body weight but not underweight are most preferred by women as partners. This finding is in line with previous studies on men’s attractiveness and BMI, which found that the most preferred male bodies resemble BMI around 21 kg/m^2^ (Swami and Tovée, 2005, 2008). The findings are also consistent with one prior study, which found an inverted U shape relationship between men’s body attractiveness and muscularity (Frederick and Haselton, 2007). Men with medium levels of muscle mass were rated to be more sexually desirable compared with the very low or very high levels of muscularity (Frederick and Haselton, 2007).

By contrast, our findings are less consistent with recent findings that stronger men are seen as more attractive (Sell et al., 2017; Foo et al., 2018) with a linear increase in attractiveness reported for the range of men’s strength sampled. There are two possible reasons for the inconsistency. Firstly, it should be noted that the studies mainly focused on attractiveness of men’s bodies rather than men’s faces. There might be a discrepancy between the attractiveness of men’s bodies and faces. Women might find a stronger body attractive but not necessarily the face shape accompanying such a body. Future study may set out to test whether women show consistent preferences for men’s body muscularity and the facial correlates of muscle.

Second, the studies that found a positive relationship between strength and attractiveness have adopted a correlational method comparing strength to ratings of natural bodies (Sell et al., 2017; Foo et al., 2018), while we employed an interactive method to let participants optimize the most attractive face shape from stimuli synthesized with computer graphics. Support for the divergence of results reflecting different methods comes from the study of Brierley et al. (2016) who used a similar interactive method to test the attractiveness of men’s bodies. Brierley et al. (2016) found that a slight decrease of body fat and slight increase of body muscle was optimal for men with normal starting BMI and body composition. In both the experiment of Brierley et al. (2016) and the experiment here, men with a high muscular body composition were not the most attractive. Studies comparing ratings of real and computer-manipulated images may help resolve the difference in attraction of strong and muscular men.

Although our hypotheses are supported with the use of both 2D and 3D facial stimuli, we note that a higher BMI (in both fat and muscle dimensions) was preferred in 3D faces compared to 2D faces. This effect of dimensionality might be due to the fact that our 3D stimuli combined both the front and the profile views, whereas our 2D stimuli used the front view alone. The combination of front and profile views may provide more information relating to weight. Alternatively, the profile view may provide information that is distinct from that evident in the front view. Indeed, prior study has shown that women make different choices for attractiveness and dominance when viewing front and profile views of the male faces (Swaddle and Reierson, 2002). Furthermore, Danel et al. (2018) showed that the measured sexually dimorphic facial features show only a moderate correlation across front and profile views (*r* = 0.20). These findings imply that further experiments are required to understand the processing of frontal and lateral views of the face.

## Conclusion

In summary, we have shown the distinct effects that facial correlates of fat mass and muscle mass have on perceptions of masculinity and attractiveness in men. Our findings show that the facial correlate of muscle mass has a profound impact on perceived facial masculinity in men of all weights. By contrast, the facial correlate of fat mass affects masculinity only in underweight to lower normal weight men. Further, we find a contextual shift in women’s attraction to the facial correlate of muscle mass but not fat mass, with a stronger preference for male face shapes associated with high muscle mass under a short-term relationship context compared to a long-term relationship context.

Body size has an impact on a variety of social judgments including attractiveness, strength, dominance, leadership and employment (Windhager et al., 2011; Re and Perrett, 2014; Holzleitner and Perrett, 2016; Nickson et al., 2016; Phalane et al., 2017). Our findings highlight the importance of differentiating size-related effects separately for body fat and body muscle.

In spite of consistent results across the three face sets and two samples of participants, we note that the current studies used a limited number of face identities that were restricted to Caucasian ethnicity. A large and more diverse sample of faces should be employed in future studies.

## Author Contributions

XL and DP conceived and designed the studies, analyzed the data, and wrote the manuscript. XL contributed to 2D stimuli production and data collection. IH and XL contributed to 3D stimuli production.

## Conflict of Interest Statement

The authors declare that the research was conducted in the absence of any commercial or financial relationships that could be construed as a potential conflict of interest.

## Footnotes

1 http://users.aber.ac.uk/bpt/jpsychomorph/
