# Supplementary material for: The Influence of Body Composition Effects on Male Facial Masculinity and Attractiveness
Source: Front Psychol. 2019 Jan 4;9:2658. doi: 10.3389/fpsyg.2018.02658 (PMC6328455; doi:10.3389/fpsyg.2018.02658)
Supplement: Supplementary file 1 [file Table_1.docx]

**Supplementary Materials**

Tabulation details for prototypes

**Table 1**. Differences of anthropometric data in high and low fat and muscle mass prototypes (3D face set and 2D version of 3D face set)

| Dimension manipulated | Dimension | Low mean | High mean | Mean difference |
| --- | --- | --- | --- | --- |
| Fat | Fat | 4.2kg | 15.3kg | 11.1kg*** |
|  | Muscle | 60.8kg | 61.9kg | 1.1kg(n.s.) |
|  | Height | 180.4cm | 180.1cm | 0.3cm(n.s.) |
|  | Age | 20.7 | 21.8 | 1.1(n.s.) |
|  | BF% | 6% | 19% | 13%*** |
|  | BMI | 20.9 | 24.8 | 3.9*** |
| Muscle | Fat | 8.1kg | 8.8kg | 0.7kg(n.s.) |
|  | Muscle | 57.3kg | 65.5kg | 8.2kg*** |
|  | Height | 181.7cm | 180.3cm | 1.4cm(n.s.) |
|  | Age | 21.0 | 20.2 | 0.8(n.s.) |
|  | BF% | 12% | 11% | 1%(n.s.) |
|  | BMI | 20.8 | 23.8 | 3.0*** |

*Note.* BMI = Body Mass Index, BF% = Body Fat Percentage

*** p<.001

n.s. (non significant)

**Table 2**. Differences of anthropometric data in high and low fat and muscle mass prototypes (independent 2D face set)

| Dimension manipulated | Dimension  measured | Low mean | High mean | Mean difference |
| --- | --- | --- | --- | --- |
| Fat | Fat | 3.8kg | 18.9kg | 15.1kg*** |
|  | Muscle | 66.8kg | 68.1kg | 1.3 kg(n.s.) |
|  | Height | 184.1cm | 183.6cm | 0.5 cm(n.s.) |
|  | Age | 20.5 | 20.8 | 0.3(n.s.) |
|  | BF% | 5% | 21% | 16%*** |
|  | BMI | 21.9 | 26.8 | 4.9*** |
| Muscle | Fat | 7.3kg | 7.9kg | 0.6kg(n.s.) |
|  | Muscle | 57.4kg | 67.7kg | 10.3kg*** |
|  | Height | 182.8cm | 182.9cm | 0.1cm(n.s.) |
|  | Age | 22.3 | 23.3 | 1.0(n.s.) |
|  | BF% | 11% | 10% | 1%(n.s.) |
|  | BMI | 20.3 | 23.7 | 3.4*** |

*Note.* BMI = Body Mass Index, BF% = Body Fat Percentage

*** p<.001

n.s. (non significant)
