# Supplementary material for: The Influence of Body Composition Effects on Male Facial Masculinity and Attractiveness
Source: Front Psychol. 2019 Jan 4;9:2658. doi: 10.3389/fpsyg.2018.02658 (PMC6328455; doi:10.3389/fpsyg.2018.02658)
Supplement: Supplementary file 2 [file Table_2.docx]

**Masculinity data**

The numbers are average masculinity ratings across the same stimuli type given by each participants. Data labels are separated into three parts by the underline.

1. The first part refers to the dimension that the faces were transformed. “FAT” means faces were transformed on the fat dimension. “MUSCLE” means faces were transformed on the muscle dimension.

2. The second part refers to the prototypes being used to transform faces. “3D” means 3D faces being transformed with the 3D prototypes. “2D(3Dmatched)” means 2D faces being transformed with the matched 2D version of 3D prototypes. “2D(independent” means 2D faces being transformed with the independent 2D prototypes.

3. The last part refers to the levels faces being transformed. “-4” means faces were transformed to lose fat/muscle by 4 BMI units. Similarly, “-2.3” means reduction in BMI by 2.3 units. “0” means no change at all. “2.3” means increase in BMI by 2.3 units. “4” means 4 BMI units increase.

**Mturk preference data and Students preference data**

These two data sets refer to women’s preference for men’s facial attractiveness. The numbers represent the associated BMI of the faces across face identities.

The structure is basically the same. The first 12 columns are the main variables used to do ANOVA analysis. Data labels are separated into three parts by the underline.

1. “long” and “short” refer to women’s choice for long-term relationships and short-term relationships.

2. “fat” and “muscle” refer to faces being transformed on fat or muscle dimension.

3. “3D” means 3D faces being transformed with the 3D prototypes. “2D(3Dmatched)” means 2D faces being transformed with the matched 2D version of 3D prototypes. “2D(independent” means 2D faces being transformed with the independent 2D prototypes.

The rest variables are average of some of the first 12 variables. They are used to do paired-samples t-tests. For example, “long_fat_average” means average scores across the faces transformed with three different prototypes on fat dimension for long-term relationship task.
