# Supplementary material for: The Influence of Body Composition Effects on Male Facial Masculinity and Attractiveness
Source: Front Psychol. 2019 Jan 4;9:2658. doi: 10.3389/fpsyg.2018.02658 (PMC6328455; doi:10.3389/fpsyg.2018.02658)
Supplement: Supplementary file 3 [file Table_3.docx]

Web address for downloading R script

http://www.perceptionlab.com/downloads/bodyComposition_v1.1.html
